# TRP Channels as Sensors of Bacterial Endotoxins

**DOI:** 10.3390/toxins10080326

**Published:** 2018-08-11

**Authors:** Brett Boonen, Yeranddy A. Alpizar, Victor M. Meseguer, Karel Talavera

**Affiliations:** 1 Laboratory for Ion Channel Research, Department of Cellular and Molecular Medicine, KU Leuven, VIB Center for Brain & Disease Research, O&N1 Herestraat 49 — box 802, 3000 Leuven, Belgium; brett.boonen@kuleuven.vib.be (B.B.); yeranddy.aguiaralpizar@kuleuven.vib.be (Y.A.A.); 2 Instituto de Neurociencias de Alicante, Universidad Miguel Hernández-CSIC, 03550 San Juan de Alicante, Spain; vmeseguer@umh.es

**Keywords:** LPS, TRPA1, TRPV4, sensory neurons, epithelial cells

## Abstract

The cellular and systemic effects induced by bacterial lipopolysaccharides (LPS) have been solely attributed to the activation of the Toll-like receptor 4 (TLR4) signalling cascade. However, recent studies have shown that LPS activates several members of the Transient Receptor Potential (TRP) family of cation channels. Indeed, LPS induces activation of the broadly-tuned chemosensor TRPA1 in sensory neurons in a TLR4-independent manner, and genetic ablation of this channel reduced mouse pain and inflammatory responses triggered by LPS and the gustatory-mediated avoidance to LPS in fruit flies. LPS was also shown to activate TRPV4 channels in airway epithelial cells, an effect leading to an immediate production of bactericidal nitric oxide and to an increase in ciliary beat frequency. In this review, we discuss the role of TRP channels as sensors of bacterial endotoxins, and therefore, as crucial players in the timely detection of invading gram-negative bacteria.

## 1. Introduction

Immune cells detect infection via specialized pattern recognition receptors that recognize pathogen- and damage-associated molecular patterns (PAMPs and DAMPs). PAMPs are typically pathogen components that have functions key for survival and the DAMPs are factors, such as ATP, that are released upon the tissue injury caused by the invading pathogens. Lipoteichoic acids (LTA) and lipopolysaccharides (LPS) are essential building elements of the wall of gram-positive and gram-negative bacteria, respectively. These components are released upon bacterial division and lyses and are detected by mammalian cells via Toll-like receptors (TLR) 2 and 4, triggering immune inflammatory responses that eventually clear and kill the invading pathogens [1].

However, recent evidence shows that the detection of invading bacteria does not rely entirely on the signalling mechanisms of pattern recognition receptors expressed in immune cells. Indeed, some bacterial components, such as *N*-formylated peptides and the pore-forming toxin α-hemolysin produced by gram-positive bacteria, can elicit Ca^2+^ influx and action potential firing in mouse nociceptive neurons [2].

Particular attention has been given to LPS, which are the most important molecules for the sensing of gram-negative bacteria [3]. The TLR4 complex is described as the key cellular component for the recognition of LPS [4]. In addition to inflammation, bacterial infections are accompanied by somatic or visceral pain. The generation of these symptoms was generally attributed to the activation of nociceptors secondary to immune activation [5]. However, neuronal activity in the vagal ganglia was shown to occur before the intercellular signalling cascades of the immune system had the time to mature [6]. Immunohistochemical analyses showed a capsaicin-sensitive subclass of trigeminal nociceptors that express TLR4, making sensory neurons probable direct targets of bacterial components [7,8]. The interaction of LPS with TLR4 expressed in rat trigeminal neurons was found to sensitize the capsaicin receptor TRPV1, resulting in release of calcitonin gene-related peptide (CGRP) [9,10]. However, dorsal root ganglion (DRG) neurons could still be excited in the absence of a functional TLR4 [11]. Although TLR4 is expressed in nociceptive sensory neurons, its role in excitability remains unclear as often its enhancing nociceptive action depends on an indirect mechanism based on sensitization of TRPV1, instead of direct triggering of action potentials. In that sense, responses to capsaicin in sensory neurons from *Tlr4* knockout mice were found to be reduced, while the effect of agonist allyl isothiocyanate (AITC), an agonist of the noxious chemical sensor TRPA1, remained unaltered [12]. Additionally, increases in intracellular Ca^2+^ concentration in rat DRG neurons, shortly after LPS challenge, were proven necessary for subsequent CGRP release [13]. Furthermore, hyperalgesia induced by intraplantar injection of LPS was reduced by a TRPA1 inhibitor and in *Trpa1* knockout mice. This effect was proposed to be related to hydrogen sulphide, which activates TRPA1, as it was prevented by blocking the production of this compound [14].

While long-term neuron-related symptoms triggered by LPS are readily explained by activation of TLR4-mediated pathways, the mechanism underlying the acute responses remained unidentified. TRP channels play an increasingly acknowledged role in inflammatory responses, not only through the phenomenon of neurogenic inflammation but also via their function in endothelial cells, pro-inflammatory immune cells and epithelial cells. Here, we review recent studies unveiling TRP channels as newly-described players in LPS recognition and acute neuronal and non-neuronal (epithelial) effects, in mammals as well as fruit flies.

## 2. TRP Channels: Structural Features and Expression Profile

**Figure 1:**
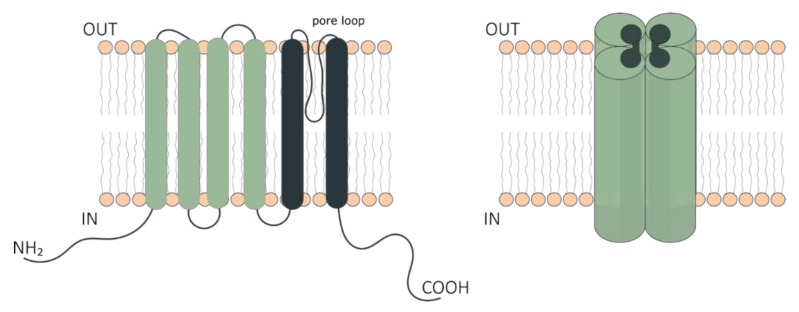
Schematic representation of Transient Receptor Potential (TRP) ion channel structure. The six-transmembrane segment topology of the monomer (**left**) and tetrameric functional unit of TRP channels (**right**). The cytoplasmic NH_2_- and COOH-terminal domains, and the transmembrane domain constituting the pore region are indicated. The transmembrane domain comprises segments 1–4 (green) distal to the pore and the segments 5 and 6 (dark green) proximal to the pore of the channel.

The TRP ion channel family consists of 13 to 28 members depending on the species [15,16]. Mammals have 28 members divided in six subgroups according to amino acid sequence homology [17]: TRPC (canonical), TRPV (vanilloid), TRPM (melastatin), TRPP (polycystin), TRPML (mucolipin), TRPA (ankyrin). Despite variations in sequence homology, TRP channels share a similar architecture composed of identical or homologous tetramers [18], in which each subunit contributes to a shared selectivity filter and ion-conducting pore [19]. Each of the four subunits is composed of a six transmembrane (TM) domain, with relatively long intracellular carboxy (C-) and amino (N-) termini containing several regulatory modules or motifs that can vary significantly between the different subfamilies [20,21]. Examples of functional domains found in TRPs are coiled coils, calmodulin-binding sites, lipid-interaction domains, EF hands and phosphorylation sites [22,23] (Figure 1). Recent cryo-electron microscopy studies revealed a high-resolution view on the structures of these channels [24].

TRP channels are non-selective cation channels, meaning that their activation results in immediate Na^+^ influx and K^+^ efflux, which tends to null the resting membrane potential of the cells [15]. Most TRP channels (except TRPM4 and TRPM5) also permeate Ca^2+^, and therefore constitute key gateways for this important regulator of multiple intracellular signalling processes. Virtually every cell in the body expresses at least one member of the TRP channel family. Because of their widespread localization, TRP channels contribute to essential physiological processes, ranging from pure sensory functions (such as pheromone signalling, taste transduction, nociception and temperature sensation) and homeostatic functions (such as Ca^2+^ and Mg^2+^ reabsorption and osmoregulation) to motile functions (e.g., muscle contraction and vasomotor control) [16,22,25,26]. In nociceptive neurons, the activation of TRP channels leads to action potential firing generating pain. Ca^2+^ influx through TRP channel activation initiates intracellular signalling pathways altering membrane protein function and triggers the release of signalling peptides [27,28,29]. TRPV1 and TRPA1 have been the most studied TRP channels in the field of neurogenic inflammation. The functional expression of these channels in nociceptors and their contribution to neurogenic inflammation are clearly established (reviewed in Reference [30]).

TRP channels are notable for being polymodal sensors, meaning that they are gated by multiple types of stimuli. Several TRP channels are sensitive to temperature changes and are hence dubbed thermoTRPs [31]. In mammals, these include TRPV1-4, TRPM2-5, which are heat-sensitive [32,33,34,35,36,37,38,39,40], and TRPM8, TRPA1 and TRPC5 are activated by cooling [41,42,43]. The ability to detect variations in temperature, pressure and chemical composition in the nerve terminal environment indicates that TRP channels function as molecular integrators of multiple sensory modalities. Finally, the coexpression of TRP channels and neuropeptides in the same sensory nerves suggests that the activation of several TRP channels may drive mechanisms that result in protective and/or detrimental processes triggered by neurogenic inflammation [44].

### 2.1. TRPA1

TRPA1 is a Ca^2+^-permeable non-selective cation channel with reported values of P_Ca_^2+^/P_Na_^+^ between 0.8 and 7.9 [45,46,47]. This channel is activated by a large number of irritants found in plants, food, cosmetics and pollutants [48]. Several TRPA1 agonists are highly reactive electrophiles with a shared ability to modify covalently cysteine and lysine residues in the N-terminus of the channel [49,50]. Examples of electrophilic activators are allyl isothiocyanate (found in wasabi and mustard), cinnamaldehyde (in cinnamon), nicotine (in formulations of smoking cessation therapies), and acrolein (in exhaust gasses) [51,52,53,54]. TRPA1 functions as a chemosensor in sensory tissues involved in innate defence mechanisms, thereby contributing to the organism’s integrity [55,56]. Activation of TRPA1 in sensory neurons produces acute pain [52], thermal and mechanical hyperalgesia, and more general, inflammatory pain [51]. As such, TRPA1 is involved in various molecular pathways leading to inflammatory pain.

TRPA1 is expressed in numerous tissues throughout the body. Sensory neurons of the vagal, nodose, trigeminal and dorsal root ganglia express TRPA1 in unmyelinated C-fibers and myelinated Aδ-fibers, both in peptidergic and non-peptidergic neurons. TRPA1 expression was also reported in non-excitable cells, such as epithelial cells, fibroblasts [57], pancreatic islet cells, several cell types in the gastrointestinal tract [58,59,60], vascular endothelial cells [61] and several cell types in the respiratory tract [62,63]. This heterogeneous distribution of TRPA1 suggests its potential implications in physiological functions and pathological conditions, within and beyond the nervous system.

### 2.2. TRPV1

TRPV1 was first identified as the receptor of capsaicin, the main pungent component of chili peppers, and as a heat sensor [64]. Capsaicin is highly specific for TRPV1 [65,66], acting as a gating modifier of the channel by shifting the voltage dependence of activation to more negative potentials, thereby increasing the open probability of the channel at the resting potential of sensory neurons [67,68,69]. TRPV1 has a relatively high permeability for Ca^2+^, with a P_Ca_^2+^/P_Na_^+^ between 9.6 and 10 [70]. TRPV1 activation by capsaicin induces rapid influx of Ca^2+^ and causes channel desensitization. This effect made capsaicin an attractive compound for medicinal use, as it renders the channel unable to re-open and in such a way causes an analgesic effect [71]. In addition, TRPV1 can be activated by heat [64], protons [72,73], and many chemical compounds (reviewed in Reference [48]). Because of its wide spectrum of chemical and physical sensitivities, TRPV1 is a main example of integrative molecular sensor of the TRP superfamily.

TRPV1 is expressed in different tissues throughout the body. Highest expression levels are found in sensory neurons of the nodose, trigeminal and dorsal root ganglion neurons [74], specifically, in small and medium diameter neurons [75]. TRPV1 expression has also been reported in brain, liver, granulocytes and mast cells [16,23,76,77,78].

In vivo activation of TRPV1 is associated with a sharp and burning pain, perceived as pungency. The polymodal sensitivity of TRPV1, and its presence in sensory neurons validates TRPV1 function in pain sensation, with a significant role in inflammatory hyperalgesia [36,65,71,79].

### 2.3. TRPV4

TRPV4 can be activated by diverse stimuli including moderate heat, cell swelling and endogenous and exogenous ligands [34,80,81,82,83,84,85,86,87,88,89]. TRPV4 is a moderate Ca^2+^ selective ion channel, with a P_Ca_^2+^/P_Na_^+^ ranging between 6 and 10 [80,82,90]. TRPV4 expression has been reported in various neuronal tissues: neurons of the hippocampus, sensory ganglia, the myenteric plexus of the colon, and sympathetic and parasympathetic nerve fibres [91,92]. In addition, TRPV4 is functionally expressed by different subsets of glial cells, astrocytes and microglia in the CNS, satellite glia that ensheath the somata of sensory neurons and Müller glia in the retina [93,94,95,96].

In non-neuronal cells, TRPV4 functions as enhancer of neuronal excitability of nearby neurons, modifying inflammatory pathways by modifying the secretion of chemokines and other factors, and osmotic homeostasis [94]. TRPV4 is an essential contributor to inflammation-induced mechanical hyperalgesia and several types of neuropathy [97]. TRPV4 activity in vascular endothelial and smooth muscle cells, keratinocytes and fibroblasts promotes diverse systemic functions including vasodilation, barrier function, wound healing, bladder voiding and ciliary beating frequency [98]. TRPV4 activation is sensitized downstream stimulation of PAR2, histamine and serotonin receptors [99,100]. Intriguingly, the functional deletion of TRPV4 in mice does not lead to any obvious sensory, muscular or inflammatory phenotypes [97]. Yet, multiple human TRPV4 mutations are linked to phenotypically divergent inflammatory, metabolic and musculoskeletal disorders [101]. The fact that TRPV4 plays a fundamental and quite wide-ranging role in normal physiology makes it a fascinating, yet complex potential pharmacological target [97,102].

## 3. TRP Channels Are Sensors of Bacterial Endotoxins

### 3.1. Sensory TRP Channels as Effectors of Pathogen-Derived Cues

The first study involving endotoxin recognition by TRP channels, led to the discovery of TRPA1 as an endotoxin sensor [103]. It was shown that *Escherichia coli* LPS activates mouse sensory neurons isolated from nodose and trigeminal ganglia. LPS activation in sensory neurons was independent of TLR4, but was rather relying on functional TRPA1 expression. Additionally, several acute LPS responses in mice are mediated by TRPA1, including hyperalgesia, tissue swelling, CGRP release and arterial dilation. In short, this study firmly established the role of TRPA1 as an LPS sensor, triggering acute responses such as pain and shaping the inflammatory outcome.

As mentioned before, sensory nerves express several members of the TRP ion channel family, whether or not together with TRPA1, which are known to play notable roles in neurogenic inflammation. For instance, TRPV1 and TRPM3 expression in sensory neurons partially overlaps with that of TRPA1 and both former channels are involved in neurogenic inflammation and the release of peptidergic modulators upon stimulation. Additionally, TRPM8 expression in sensory neurons is linked to cold hyperalgesia in inflammatory states [104,105,106].

**Figure 2:**
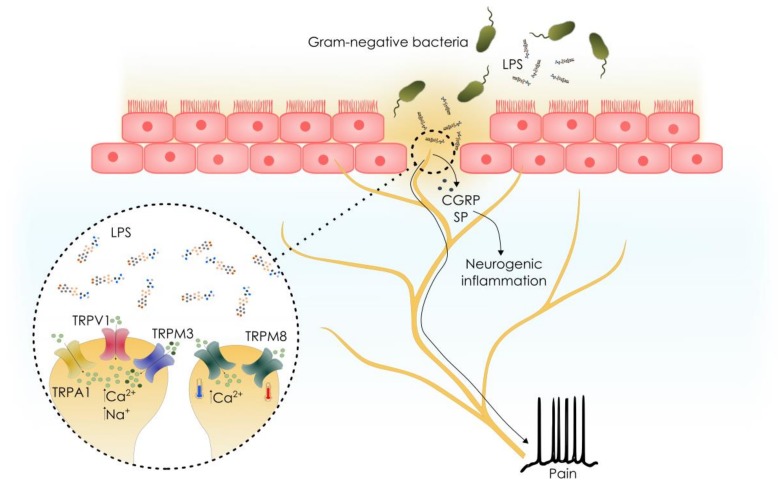
Effects of the activation of TRP channels in sensory neurons by lipopolysaccharides (LPS). LPS triggers activation the sensory nervous system by activation of TRPA1, TRPV1 and/or TRPM3 located at peripheral nerve terminals. TRPM8 activation by LPS occurs at cold temperatures. Sensory nerve activation leads to action potential generation and pain, and the local release of neuropeptides leading to neurogenic inflammation. CGRP, calcitonin gene-related peptide; SP, substance P.

A recent study demonstrated that LPS responses in primary cultured mouse DRG neurons are not solely mediated by TRPA1 activation [107]. This led to the discovery of LPS activity on three other TRP channels: TRPV1, TRPM3 and TRPM8 (Figure 2). Among these, TRPV1 was demonstrated to be an important contributor to the increase in intracellular Ca^2+^ concentration triggered by LPS in primary cultured mouse DRG neurons. TRPV1 activation in heterologous cells was robust and the combined ablation of *Trpv1* and *Trpa1*, almost completely abolished responses in primary cultured mouse DRG neurons. Activation of TRPV1 in nociceptive neurons is known to induce a painful burning sensation, and the release of neurogenic factors such as CGRP and substance P, leading to neurogenic inflammation. TRPV1 activation may therefore account for the previously described residual nocifensive and inflammatory effects in *Trpa1* KO mice [103]. Whole-cell patch-clamp experiments revealed that LPS readily activates TRPM3 in the heterologous expression system HEK293T. However, the weak responses of TRPM3-expressing DRG neurons extracted from *Trpa1/Trpv1* double KO mice to LPS suggest a minor role for TRPM3 in LPS detection. Whereas TRPM3 activity is implicated in inflammatory pain [33,108], no available data indicates a role in pathogen sensing. Although the direct effect of LPS on TRPM3 is limited, the expression pattern of this channel in nociceptors overlaps with TRPA1 and TRPV1, and therefore, sensitization or synergistic effects with TRPA1, TRPV1 or TLR4-mediated signalling should be investigated in future research.

LPS responses in TRPM8-transfected cells were strongly modulated by temperature. At 35 °C, responses to LPS were virtually indistinguishable from non-transfected cells, indicating that TRPM8 does not contribute to neuronal responses to LPS in normal physiological temperatures. In contrast, a large proportion of TRPM8-expressing cells responded to LPS, in a dose dependent manner at 25 °C.

The contribution of TRPM8 activation in inflammatory processes is paradoxical. Topical menthol application and cold compresses alleviate pain, whereas cold air can induce coughing, and TRPM8 ablation sometimes attenuates and sometimes aggravates inflammatory conditions [109,110]. Additionally, not all TRPM8-expressing tissues are exposed to environmental temperature fluctuation. Thus, further research will be required to determine whether the temperature dependence of TRPM8 activation by LPS is relevant for LPS signalling.

Although the mechanism of TRP activation by LPS remains elusive, several indications suggest that TRPA1 can detect LPS-induced membrane perturbations. For instance, LPS-induced activation of TRPA1 currents in isolated membrane patches precludes the necessity of soluble intracellular mediators. Notably, the availability to the lipid A was shown to be both necessary and sufficient for TRPA1 activation and slight changes in the chemical (lipid A) structure of LPS between different bacterial strains resulted in distinct efficacy for TRPA1 activation [103]. Furthermore, the inflammatory outcome induced by LPS derived from different bacterial species correlated with the degree of TRPA1 activation. While further research is required, in order to clarify the interactions between LPS, the lipid bilayer and TRPA1, the proposed mechano-sensory properties of TRPA1 accommodate well such mechanism [56,111,112,113].

Regarding the activation of TRPV1 by LPS, also the underlying mechanism has yet to be identified. One may hypothesize that LPS modulates TRPV1 analogously to other endogenous lipid modulators, such as DAG, PIP_2_, metabolites of arachidonic acid and lysophosphatidic acid (LPA) [114,115]. However, this does not seem to be the case. TRPV1 activation by the lipid second messenger DAG depends on an intact S513 residue [116], yet this mutation did not abrogate LPS-induced TRPV1 activation [107]. This also excludes secondary activation due to TLR4-mediated DAG production (see Section 2.3). Neither is there a necessity for C157, a residue required for electrophile activation [117]. Activation of TRPV1 by LPA occurs by electrostatic interaction with positive charges within the proximal C-terminus of the channel with LPA present in the inner leaflet of the plasma membrane [118,119]. While several studies indicate that LPS can insert in the outer leaflet of the membrane [120,121], due to its spatial dimensions it is unlikely that flipping mechanisms accommodating the large *O*-polysaccharide operate in mammalian cells. Alternatively, LPS could be taken up by the cell and reach the inner leaflet of the membrane via an endocytosis pathway [122]. Nevertheless, these processes take time and activation of TRPV1 by LPS occurs within seconds. Thus, acute LPS activation of TRPV1 seems to work via a mechanism different from those known for other lipid mediators.

### 3.2. TRPA1 is A Conserved Bacterial Endotoxin Sensor in Drosophila

TRPA1 function as a chemosensor is strongly conserved in many animals, including *Drosophila melanogaster* fruit flies [55]. These flies live and breed in microbe-rich food substrates, such as rotten fruits. Therefore, the ability to detect and interpret a wide variety of noxious chemicals amid a cacophony of chemical noise may be a matter of life and death. Fruit flies detect dangerous substances in the environment via chemoreceptors expressed throughout their body [123]. LPS is non-volatile in nature, and upon contact with peripheral gustatory receptor neurons (GRNs), LPS induces grooming behaviour in flies. LPS-induced grooming depended on taste receptors in bitter-sensing GRNs in the legs and wings of the fly and persisted in decapitated flies [124]. While LPS-induced grooming was absent in flies lacking peripheral gustatory receptors, the molecular LPS sensor in the legs and wings remains to be identified. In addition, female flies avoid LPS contaminated food for oviposition [125].

Oviposition aversion for LPS depends on a small subset of GRNs containing both *dTrpA1* and a gustatory receptor for bitter compounds, *Gr66a*, located in the proboscis of the fly [125]. Even though *Gr66a*-positive GRNs are present in chemosensory tissues throughout the body of the fly, *dTrpA1* expression overlap within those tissues is restricted to the GRNs in the labellum and labral sense organ (LSO) [126]. The GRNs on the labellum probe the food prior to ingestion, resulting in an extension of the proboscis of the fly to attractive chemicals and a reduction of this reflex in the presence of aversive tastants [127]. Since the proboscis extension reflex to sucrose was unaltered in the presence of LPS, the avoidance behaviour is mediated by the GRN activity in the LSO, after food ingestion. In the insect gustatory system, bitter-sensing GRNs express several chemoreceptors and dTRPA1 is known to function as a crucial downstream component of other chemosensors implicated in behavioural avoidance [128]. It would be of interest to investigate whether *dTrpA1* is involved in LPS grooming behaviour triggered by the tarsal or wing GRNs. In conclusion, these reports demonstrate that fruit flies possess two gustatory mechanisms underlying the detection of LPS, one of which is heavily dependent on TRPA1 expression.

LPS sensitivity in the fly is not restricted to peripheral sensory tissues. Even neurons of the larval CNS detect and respond to LPS [125], a sensory feature that might be explained by the presence of *dTrpA1* in certain regions of the brain [126]. A subsequent study showed that exposure to the gram-negative bacterium *Erwinia corotova* (Ecc15) causes feeding cessation in larvae of *D. melanogaster* and *Drosophila suzukii* [129]. Further genetic exploration in *D. melanogaster* determined that feeding cessation was independent of uracil content and depending on *dTrpA1* and *orco*, indicative of an olfaction-based mechanism. This behavioural immunity represents a first line of defence that can prevent the animal from being in contact with a microbe or limit the duration of the contact, thereby reducing the risk of being infected.

TRPA1 activation has been reported to trigger many complementary protective mechanisms. For instance, the activation of dTRPA1 isoforms C and D in *Gr66a*-positive neurons in the proboscis causes avoidance of egg laying female flies to UV and blue light [130]. Likewise, Du et al. reported an increase in the intestinal transit resulting from dTRPA1(C) activation by reactive oxygen species (ROS) produced in response to uracil secretion by organisms including Ecc15 [131]. In addition, oxidative stress in the fly gut promotes intestinal stem cell proliferation and self-renewal depending on Ca^2+^ signalling events which rely on functional expression of dTRPA1(D) [132]. Both processes were proposed as defence mechanisms against oxidative stress insults. The fact that dTRPA1 is implicated in these sensory mechanisms suggests a broadly conserved principle whereby this channel play a crucial role in LPS detection by sensory neurons in flies and mammals, regardless of the particular sensory modality involved. The ancient nature of LPS and the conserved role of TRPA1 in chemical avoidance from flies to mammals underscore the evolutionary significance of this mechanism in animals.

### 3.3. Role of Non-Neuronal TRP Channels in LPS Detection

The epithelial barrier, both mucosa and the epidermis of the skin, is composed by highly specialised stratified epithelia protecting the body from dehydration and heat loss. Located at the interface between the environment and the host, it also protects against physical and chemical damage, and importantly, against invading pathogens [133,134,135]. This critical barrier function is maintained through diverse mechanisms, in which TRP channels play crucial roles.

In the skin, the formation of cell-cell junctions in differentiating keratinocytes critically depends on TRPV4-mediated Ca^2+^ influx [136]. Located at the adherens junctions, TRPV4 directly interacts with β-catenin and E-cadherin, thereby triggering Ca^2+^-dependent re-arrangement, and stratification of actin fibres and the formation of tight junctions and adherens junctions at the apical contact side of stratified cells [136,137,138]. This results in strengthened cell-cell adhesion structures between keratinocytes and the basal membrane [133], thus forming a physical barrier against environmental insults.

TRPV4 channel function is also essential in the regulation of ciliary beat frequency (CBF) of ciliated epithelial cells in the airways. In these cells, TRPV4 expression is markedly polarised towards the apical side, at the base of the cilia and in the cilia itself, directly providing Ca^2+^ upon mechanical and chemical stimulation leading to increased CBF and mucus clearance [139,140].

**Figure 3:**
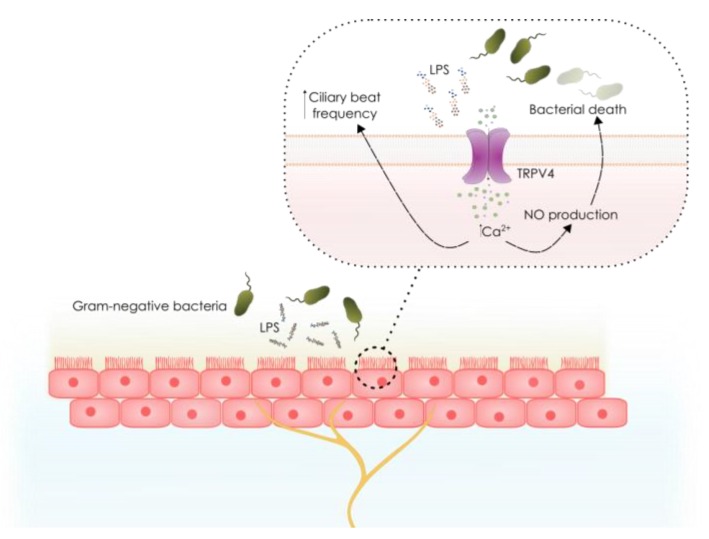
Acute responses in epithelial cells after activation of TRPV4 by LPS. LPS shed by gram-negative bacteria during colonization near the epithelial surface activates TRPV4 channels expressed in ciliated epithelial cells, leading to a Ca^2+^ influx that triggers acute cellular responses such as the production of bactericidal nitric oxide and increased ciliary beat frequency. NO, nitric oxide; CBF, ciliary beat frequency.

In airway epithelial cells, TRPV4 channel also functions as a sensor of bacterial endotoxins. LPS activates TRPV4 resulting in an acute increase in intracellular Ca^2+^, triggering a variety of immediate defensive responses [141]. TRPV4-mediated rise in Ca^2+^ induces the activation of nitric oxide synthases (eNOS and iNOS) constitutively expressed in epithelial cells, increasing nitric oxide (NO) production in the luminal compartment, resulting in a bactericidal effect (Figure 3). On the other hand, NO would presumably diffuse to the basal face of the epithelial layer, inducing bronchodilation through its relaxing action on airway smooth muscle [142,143], and decreasing leukocyte adhesion to endothelial cells [144,145,146,147,148] through down-regulation of cell adhesion molecules [149,150,151]. Similar to other TRPV4 agonists, such as heat and GSK1016790A [140], LPS-induced TRPV4 activation increases the CBF, suggesting that TRPV4 could also enhance the clearance of pathogens from the airways. Thus, TRPV4 is a locus for detection and immediate local responses against invading bacteria.

The identification of TRPV4 as LPS sensor in non-excitable cells shed new light on the mechanisms underlying the activation of TRP channels by this endotoxin. For instance, TRPV3 is also expressed in keratinocytes and epithelial cells, it is activated by mild heating (>33 °C) [152] and yet it is largely insensitive to LPS [141]. As for TRPA1 in sensory neurons [103], TRPV4 activation by LPS required a freely available lipid A moiety [141].

TRPV4 activation by LPS also occurs in the absence of the TLR4 signalling cascade, indicating that this mechanism operates in parallel to processest based on canonical pathogen recognition receptors. Interestingly, activation of TRPV4 in intestinal epithelial cells by a chemical agonist induces the production of IL-8, IP-10, MIG, and MCP-1 [153] and TRPV4-deficient epithelial cells stimulated with LPS displayed increased IL-6 and chemokine (Cxcl-1 and Cxcl-2) production [141]. Thus, TRPV4-mediated Ca^2+^ influx may play a dual role in initiating and tailoring an appropriate inflammatory response.

### 3.4. Role of TRP Channels in Other LPS Detection Pathways

Although the (possible) regulatory interaction between TLR4 and TRPV4 signalling cascades in epithelial cells remains unexplored, TLR4-mediated effects in other cell types do require a Ca^2+^ entry pathway through other TRP channel family members, namely TRPC6 and TRPM7 [154,155]. In endothelial cells, LPS binding to the TLR4-associated complex molecule CD14 [156], induces the hydrolysis of phosphatidylcholine with an ensuing production of diacylglycerol (DAG) [157,158]. Subsequently, DAG activates TRPC6, leading to an increase in intracellular Ca^2+^ concentration, resulting in non-muscle myosin light chain kinase activation of MyD88, inflammasome formation and NF- κB pathway activation [155]. Thus, together with the Ca^2+^-dependent endothelial barrier disruption [159,160,161,162,163,164,165], TRPC6-mediated Ca^2+^ increase is also implicated in inflammatory responses. Similarly, TRPC6-dependent Ca^2+^ influx linked to the TLR4/PI3K/Akt pathway promotes the activation of ERK1/2, p38, and NF-κB in bronchial epithelial cells [166]. Although direct activation of TRPC6 by LPS has not been assessed, the absence of Ca^2+^ responses in *Tlr4*-deficient lung endothelial cells [155] indicates that LPS-induced TRPC6 activation, unlike TRPA1, TRPV1 and TRPV4, is most likely entirely secondary to TLR4 ligation. Interestingly, TRPV4 is prominently expressed in endothelial cells [82,167], where it is involved in the hyper-inflammatory response and mortality associated with sepsis [168]. Altogether, this suggests that the combined activation of the TLR4- and TRPV4-mediated signalling cascades lead to the exacerbated cytokine-cocktail and vasodilation leading to septic shock. Nonetheless, future research should be conducted to elucidate the individual contribution of TRP channels in sepsis.

Endothelial cell death following LPS exposure involves early ROS production [169] and Na^+^ influx through TRPM4 [170]. Early ROS production depends on TLR4, Nox2 and Nox4 and, is suggested to be mediated by PLC/cPKC and PI3-K pathways [169]. Interestingly, TRPM4 is a Ca^2+^-activated channel [171] and early ROS production is preceded by Ca^2+^ influx [172]. The origin of the intracellular Ca^2+^ increase prompting these pathways remains elusive. The expression profiles and onsets of Ca^2+^ influx via TRPC6 or TRPV4 correspond to the onset of these processes, yet no causal link has yet been provided.

Through a different mechanism, the axis LPS-TLR4-inflammasome in macrophages also requires TRP-mediated Ca^2+^ influx. In this case, TRPM7 mediates the LPS-induced Ca^2+^ increase required for TLR4 endocytosis, IRF3 activation, NF-κB phosphorylation and nuclear translocation, and pro-inflammatory cytokine production [154]. Similar to TRPC6 in endothelial cells, membrane-anchored CD14 (mCD14), rather than TLR4, is the molecular determinant required for LPS-mediated TRPM7 activation in macrophages. Whether mCD14 participates in epithelial TRPV4 and/or neuronal TRP channel activation remains to be investigated. Interestingly, mCD14 is expressed both in epithelial cells [173] and sensory neurons [7,174], where it may facilitate LPS recognition in the vicinity of the TRP channels. However, it remains to be determined whether events following LPS-mCD14 interaction lead to or modulate TRP activation.

Of note, TRPV2 and TRPV4 channels are also found in the surface of macrophages. TRPV2 is implicated in the actin depolymerization necessary for occupancy-elicited phagocytic receptor clustering [175]. However, LPS fails to induce TRPV2-mediated Ca^2+^ influx [107], excluding a role of TRPV2 in LPS-mediated rise in intracellular Ca^2+^. Conversely, TRPV4-mediated LPS-stimulated phagocytosis of non-opsonized particles [176] and TRPV4-evoked superoxide and nitric oxide production [177] could result from direct activation of TRPV4 by LPS.

## 4. Conclusions

In addition to the canonical pathway of LPS detection mediated by TLR4, TRP channel-mediated pathways endow sensory neurons and airway epithelial cells with the ability to detect and react to bacterial endotoxins. While several TRP channels present in sensory neurons: TRPA1, TRPV1, TRPM3 and TRPM8, detect LPS, TRPV1 and TRPA1 can account for the majority of the LPS induced neuronal activation. Yet, pain and neurogenic inflammation following acute exposure to LPS was shown to depend mainly on TRPA1 activity. The TRPA1 chemosensitivity for LPS is conserved in flies, where it plays an essential role in a gustatory mechanism underlying the detection and avoidance of LPS. Other LPS detection pathways exist in *Drosophila*, however the involvement of dTRPA1 in those pathways requires closer examination.

TRPA1 activity due to LPS exposure leads to robust defensive behaviours, yet the long-term consequences on infection outcomes or surrounding non-sensory tissues remain to be investigated. Similarly, TRPV1 activation is involved in the intricate communication between sensory nerve endings, dermal non-immune and immune cells, in part by mediating the release of inflammatory modulators. The in vivo contribution of LPS-induced TRPV1 activity in tissues innervated by DRG neurons requires further study.

While TRPM3 and TRPM8 did not seem to contribute substantially to neuronal LPS responses, their role may be more prominent in non-neuronal or diseased tissue or in conjunction with other LPS-induced cellular pathways. A limitation of the current literature is that most studies reports on cell lines and animal models. However, the recent advances in stem cell-derived human sensory neurons could provide a future solution for studying these mechanisms on human systems [178,179].

In the epithelial cells lining the mucous membrane of the respiratory system, a clear protective role for TRPV4 as an endotoxin sensor was revealed. TRPV4 recognition of LPS occurs in a TLR4-independent way and induces local secretion of NO, which in turn serves as a bactericidal defence mechanism. TRPV4 endows epithelial cells the ability to rapidly respond to LPS by initiating processes that surpass the passive physical barrier role ascribed to epithelial cell linings. As such, inhibition of TRPV4 activity in early stages of colonization would be disadvantageous in protecting against pathogen invasion. It remains to be investigated whether this response can be translated to other TRPV4-expressing tissues, for instance endothelial cells.

As TRP channels and TLRs expression overlap in many tissues, it would be of interest to explore the function of dual detection mechanisms and crosstalk between intracellular signalling pathways initiated by TLR4 activation along with TRP channel activation.

The facts that the endotoxin-sensing function is found in cells of different tissues and conserved in mice and fruit flies underscore the importance of sensory TRP channels for innate defence mechanisms, in particular in reaction to LPS.
